# A rare complication of knee hematoma after genicular nerve radiofrequency ablation

**DOI:** 10.1097/PR9.0000000000000736

**Published:** 2019-04-05

**Authors:** Natalie Strand, Paolo Jorge, John Freeman, Ryan S. D'Souza

**Affiliations:** a Department of Anesthesiology, Pain Medicine Division Clinic, Phoenix, AZ, USA; b Department of Anesthesiology and Perioperative Medicine, Mayo Clinic, Rochester, MN, USA

**Keywords:** Genicular nerve, Radiofrequency ablation, Knee pain, Bleeding

## Abstract

**Background:**

Genicular nerve radiofrequency ablation (RFA) is an intervention to treat patients with chronic knee pain who have failed previous conservative, pharmacologic, and surgical interventions. Vascular complications following interventional procedures of the knee are extremely rare. A delay in diagnosis may be detrimental for the viability of the affected limb and may ultimately require amputation.

**Case Presentation:**

A 76-year-old man with a history of severe bilateral knee osteoarthritis and grade 4 chondromalacia presented to our clinic with refractory, severe bilateral knee pain and received a bilateral genicular nerve RFA. He returned 4 days later with right medial thigh pain and a magnetic resonance imaging study revealing a hematoma along the anteromedial aspect of the right distal femoral diaphysis measuring 13.3 × 4.5 × 3.0 cm. After collaboration between pain medicine and orthopedic surgery services, decision was made to treat patient conservatively with rest, compression, elevation, ice application, tramadol, and gabapentin, but with close follow-up and a low threshold to intervene with diagnostic and therapeutic angiography with embolization if bleeding worsened; he reported resolution of his pain after a 4-day and 1-month follow-up.

**Conclusion:**

This is the first report describing iatrogenic vascular injury in the knee after a genicular RFA procedure. Pain medicine physicians should be aware of the vascular anatomy of the knee, particularly paying close attention to variations after previous surgeries. Future trials should investigate modalities that minimize vascular complications including concomitant use of ultrasonography with fluoroscopy and other forms of RFA including pulsed or cooled RFA.

## 1. Introduction

Genicular nerve radiofrequency ablation (RFA) is performed to treat patients with chronic knee pain who have failed conservative, pharmacologic, or surgical treatments. Using fluoroscopic guidance, RFA is performed at the lateral superior, medial superior, and medial inferior genicular nerves, which travel in close proximity to genicular arteries.^2^ Vascular complications following interventional procedures and surgeries of the knee are extremely scarce, with only a few case reports in the literature describing iatrogenic vascular trauma.^18,23^ Delayed diagnosis may be detrimental for the viability of the affected limb and may ultimately require amputation.^24^

Although vascular injury after genicular nerve RFA has never been reported in the literature, vascular complications of genicular arteries are documented in surgical interventions including knee arthroscopies and arthroplasties. A study conducted by the Committee on Complications of the Arthroscopy Association of North America revealed only 12 cases (0.003%) of vascular injury out of 395,566 total knee surgeries.^3^ The most common reported etiology of spontaneous bleeding after knee surgery is impingement of the hypertrophic vascular synovium or fat pad,^20,21^ but less common causes include villonodular synovitis,^4^ arteriovenous fistula,^8^ and genicular artery hematoma.^12^

We report a case of vascular injury likely to the medial superior genicular artery following a genicular nerve RFA procedure. We conduct a detailed review of the literature, with the objective of identifying and analyzing types of iatrogenic vascular lesions of genicular arteries following knee surgeries and procedures.

## 2. Case Report

A 76-year-old man with a history of severe bilateral knee osteoarthritis and grade 4 chondromalacia presented to the outpatient pain clinic to receive bilateral genicular nerve RFA. He was an otherwise healthy man with a distant history of rheumatic fever and a body mass index of 23.6 Kg/m^2^. He denied a history of knee surgeries or procedures, bleeding disorders, or chronic anticoagulant or antiplatelet therapy. Diagnostic genicular nerve blocks were performed at the superior medial genicular nerve, superior lateral genicular nerve, inferior medial genicular nerve, and the suprapatellar genicular nerve in both knees about 48 days before this visit with significant alleviation of bilateral knee pain.

**Figure 1.**
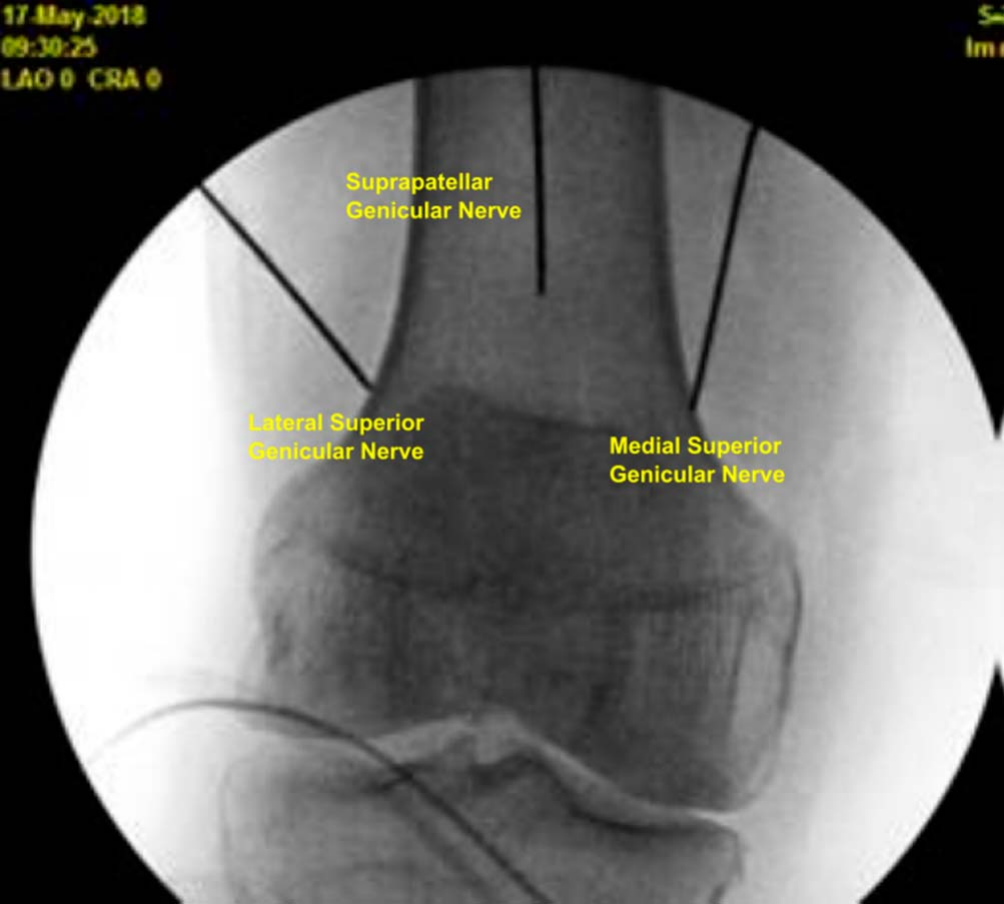
Genicular nerve RFA probe targets on anterior/posterior fluoroscopic view. RFA, radiofrequency ablation.

**Figure 2.**
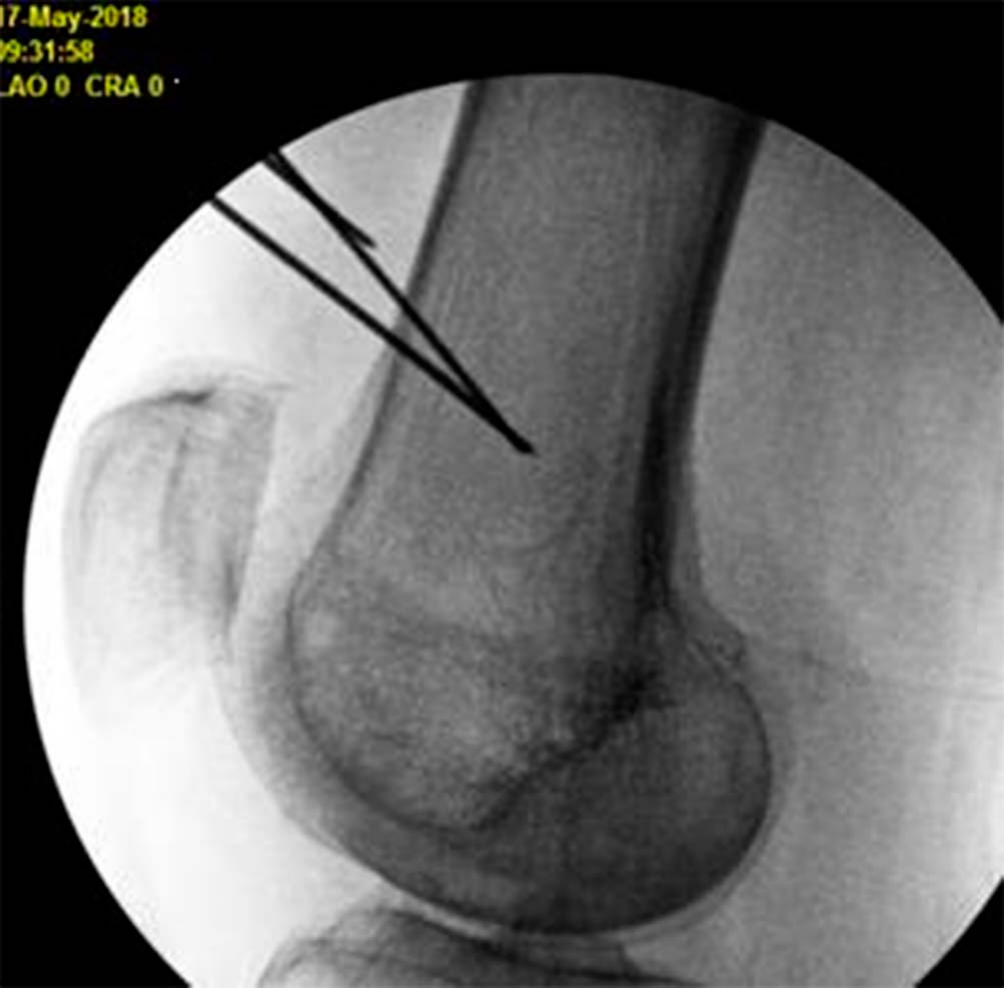
Genicular nerve RFA probe targets on lateral fluoroscopic view. RFA, radiofrequency ablation.

After local anesthetic administered, separate 17-gauge introducer needles were advanced to bony contact at the superior medial genicular nerves, superior lateral genicular nerves, inferior medial genicular nerves, and the genicular nerves 2 cm superior to the patella (Figs. 1 and 2). Lidocaine 2% was administered before lesioning with settings at 60 degrees centigrade (heating tissues to 80 degrees centigrade) for 2 minutes and 30 seconds. A total of 40-mg methylprednisolone were administered in each knee.

**Figure 3.**
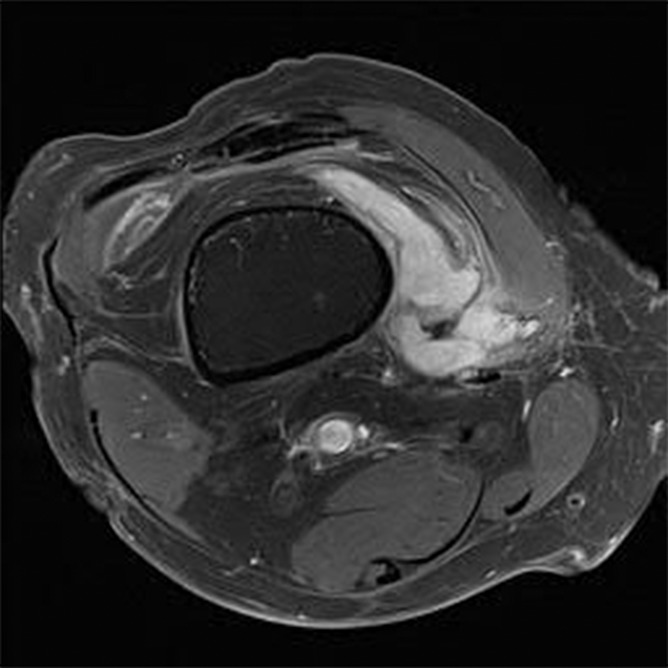
Axial T1-weighted MRI displaying peripheral nodular enhancement along the medial aspect of the distal femur likely reflecting hematoma measuring 13.4 × 4.6 × 3.0 cm. MRI, magnetic resonance imaging.

**Figure 4.**
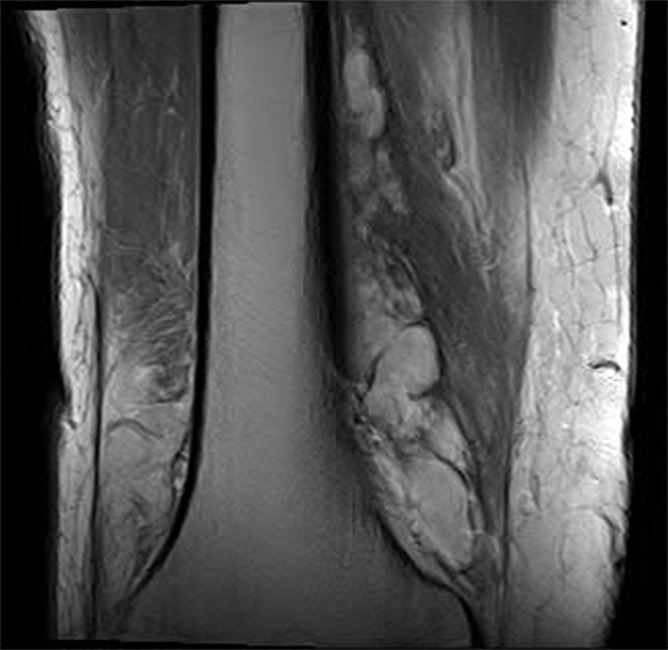
Coronal T2-weighted MRI displaying peripheral nodular enhancement along the medial aspect of the distal femur likely reflecting hematoma measuring 13.4 × 4.6 × 3.0 cm. MRI, magnetic resonance imaging.

He returned to clinic 4 days later with resolved bilateral knee pain, but significant right medial thigh pain with flexion and extension of the knee along with edema and tenderness on examination. He denied any neurological deficits, fever, fatigue, malaise, nausea, vomiting, and recent sick contacts. Coagulation studies, including platelet count, prothrombin time, and international normalized ratio, were not obtained, as the patient was otherwise healthy and denied having a history of bleeding disorders or taking anticoagulant or antiplatelet therapy. Magnetic resonance imaging (MRI) showed a hematoma along the anteromedial aspect of the right distal femoral diaphysis measuring 13.3 × 4.5 × 3.0 cm (Figs. 3 and 4). This case was discussed with the orthopedic surgery team, and they recommended conservative management, with close interval follow-up to determine whether the patient required angiography with embolization in the event of worsening hematoma. He was treated with compression, elevation, and ice. Patient was instructed to return to the emergency department if he experiencing worsening knee swelling or pain. Due to a component of suspected postablation neuritis, he was given a small supply of tramadol and started on gabapentin.

Four days later, he reported improved knee pain, and a repeat MRI revealed a stable hematoma with similar dimensions. At a follow-up visit 1 month later, he was found to be functioning well and appropriately in outpatient physical therapy.

## 3. Discussion

To the authors' knowledge, this is the first case report describing iatrogenic vascular injury in the knee after a genicular RFA procedure. Genicular nerve RFA primarily relies on bony landmarks, and usual technique may fail to provide adequate visualization of vascular structures. Vascular injury is a rare complication even following knee surgeries, particularly injury to the genicular arteries. A study by Vincent and Stanish^25^ reported only 2 cases of genicular artery injury (inferior lateral genicular and descending genicular artery) out of 2,800 knee procedures. The first report of damage to a genicular artery was made in 1987 by Manning and Marshall^16^, who reported injury to the lateral inferior genicular artery after a diagnostic arthroscopic procedure in a patient with anterior cruciate ligament rupture. Despite case reports of genicular vascular injury in the surgical literature, the etiology of injury remains unclear, but studies suggest perforation by a retractor, damage to atherosclerotic arteries, tourniquet-mediated injury, direct vessel injury, and secondary injury from cement heat (ie, during knee arthroplasty).^15^

The complexity of knee joint innervation is demonstrated by the genicular nerves arising from branches of the sciatic, femoral, and obturator nerves, all of which are derived from the lumbar plexus.^9^ Cadaveric studies reveal the lateral superior genicular nerve arises from the common peroneal division of the sciatic nerve about 8 to 10 cm above the knee joint line and travels deep to the biceps femoris and iliotibial band. The tibial nerve gives rise to the medial superior and medial inferior genicular nerves located at the medial aspect of the knee joint.^9,13^ The saphenous nerve gives off the suprapatellar and infrapatellar genicular nerves, which innervate the anterior portion of the knee.^10^ These aforementioned genicular nerves travel in close proximity to the lateral superior, medial superior, and medial interior genicular arteries.^7^ Despite comprehensively describing the anatomical locations of vasculature and innervation within the knee, cadaveric studies also highlight the high degree of variation in anatomy leading to disparities in procedural technique.^2,6,17^ Given that genicular RFA is a typically treatment for post–total knee arthroplasty pain, the pain proceduralist must be cognizant that the path taken by the genicular nerves can vary considerably among individuals and might be unpredictable due to axonal misrouting and aberrant reinnervation.^22^

A review performed by Kim et al.^13^ pooled results from 27 cases of genicular vascular injury after surgical intervention, showing that about 26% involved the lateral superior genicular artery, about 41% involved the medial superior genicular artery, and about 33% involved the medial inferior genicular artery. Most of these vascular injuries manifested as pseudoaneurysms, and less frequently as arteriovenous fistulas, hemarthrosis, and patellar osteonecrosis.^13^ A pseudoaneurysm can continue to increase in size, compressing nearby structures and potentially causing distal neurologic deficit. Rupture of the pseudoaneurysm may also manifest with significant swelling, tenderness, ecchymosis, and recurrent hemarthrosis.^11,13,21^

Vascular injury may go unnoticed for many days and even weeks. This may be due to the compressive bandaging applied at the end of a surgery or procedure.^14,16^ In addition, the improper use of Doppler with adequate signal in distal vessels may provide false reassurance.^24^ Finally, the low incidence of vascular injury predisposes the proceduralist to have a low suspicion of this complication on their differential diagnosis.

As fluoroscopic-guided genicular nerve RFA is a relatively novel procedure, long-term efficacy and data on adverse vascular complications are lacking. Regardless, the risk of vascular injury is a reasonable possibility, given the proximity of genicular vessels and nerves. It is feasible that vascular complications of genicular arteries do exist but have been underreported in the current literature, as the procedure is relatively novel. As proposed by Kim et al.,^13^ an alternative explanation could be that the sink effect of nearby blood vessels provides constant blood flow to the RFA targets,^26^ thus attenuating the lesioning temperature and leading to a superior coagulation effect than if the procedure is performed under direct needle insertion alone.

In terms of diagnostic modalities and treatment, the literature highly favors angiography and embolization, although these recommendations are anecdotal.^19,21^ Advantages with this approach include minimal exposure through percutaneous arterial catheterization of the femoral artery with decreased infection risk, combination of diagnosis and therapeutic intervention in one single procedure, no necessity for general anesthesia, and no alterations or restrictions postprocedurally in rehabilitation programs. For larger hematomas due to damage of larger vessels such as the popliteal artery, reconstruction with or without a vein graft or angioplasty patching is recommended.^14^

In our case report, although we did not pursue angiography to specifically localize the affected artery, the location of the hematoma on MRI suggests bleeding may have occurred at the medial superior genicular artery. The probable mechanism of injury may be direct shear injury to the vessel wall from radiofrequency probe insertion. We initially pursued conservative measures with rest, ice, compression, and elevation, although with close follow-up and low threshold to admit patient for further vascular intervention (eg, angiography with embolization) if his knee swelling worsened.

In conclusion, although large randomized trials are lacking on genicular RFA, this relatively novel procedure has been increasingly used among pain interventionists to treat chronic knee pain. Data on long-term efficacy and adverse outcomes are largely unavailable. Our case report emphasizes the proximity of genicular arteries to the nerves targeted by RFA and the possibility of vascular injury related to this procedure. Pain medicine physicians and orthopedic surgeons should be aware of the vascular anatomy of the knee, particularly paying close attention to variations after previous surgeries. While supportive conservative measures including rest, ice application, and elevation may treat the patient symptomatically, angiography with selective embolization is also a minimally invasive option with good outcomes. Future studies should investigate whether concomitant use of ultrasonography with fluoroscopy to visualize and avoid vasculature during genicular RFA may be associated with lower rates of vascular injury. Alternatively, pulsed or cooled RFA provides excellent alternatives that minimize the chance of tissue damage compared with conventional RFA, and studies should investigate vascular injury outcomes with these alternative approaches. In pulsed RFA, tissue damage does not occur because the temperature limit is 42°C^13^; although pulsed RFA of the sciatic nerve^5^ and saphenous nerve^1^ has been described in the treatment of chronic knee pain, future studies evaluating pulsed RFA of genicular nerves are warranted.

## Disclosures

The authors have no conflict of interest to declare.
